# An Example of Polynomial Expansion: The Reaction of 3(5)-Methyl-1*H*-Pyrazole with Chloroform and Characterization of the Four Isomers

**DOI:** 10.3390/molecules24030568

**Published:** 2019-02-04

**Authors:** Vera L. M. Silva, Artur M. S. Silva, Rosa M. Claramunt, Dionisia Sanz, Lourdes Infantes, Ángela Martínez-López, Felipe Reviriego, Ibon Alkorta, José Elguero

**Affiliations:** 1 Chemistry Department and QOPNA and LAQV-REQUIMTE, University of Aveiro, 3810-193 Aveiro, Portugal; verasilva@ua.pt; 2 Departamento de Química Orgánica y Bio-Orgánica, Facultad de Ciencias, UNED, Paseo Senda del Rey, 9, E-28040 Madrid, Spain; rclaramunt@ccia.uned.es (R.M.C.); dsanz@ccia.uned.es (D.S.); 3 Departamento de Cristalografía y Biología Estructural, Instituto de Química-Física Rocasolano, CSIC, Serrano, 119, E-28006 Madrid, Spain; angela_villalba9@hotmail.com; 4 Instituto de Química Médica, CSIC, Juan de la Cierva, 3, E-28006 Madrid, Spain; freviriegop@ictp.csic.es (F.R.); ibon@iqm.csic.es (I.A.); jelguero@iqm.csic.es (J.E.)

**Keywords:** pyrazoles, pyrazolylmethanes, phase transfer catalysis, NMR spectroscopy, X-ray crystallography, theoretical calculations, GIAO calculations

## Abstract

The reaction in phase-transfer catalyzed conditions of 3(5)-methyl-1*H*-pyrazole with chloroform affords four isomers **333**, **335**, **355** and **555** in proportions corresponding to the polynomial expansion (a + b)^3^, with a = 0.6 and b = 0.4, a and b being 3-methyl and 5-methyl proportions. The up (*u*) and down (*d*) conformation of the pyrazolyl rings with regard to the Csp^3^–H atom was established by X-ray crystallography and by ^1^H-, ^13^C- and ^15^N-NMR in solution combined with gauge-including atomic orbitals (GIAO)/B3LYP/6-311++G(d,p) calculations. A comparison with other X-ray structures of tris-pyrazolylmethanes was carried out.

## 1. Introduction

**Figure 1:**
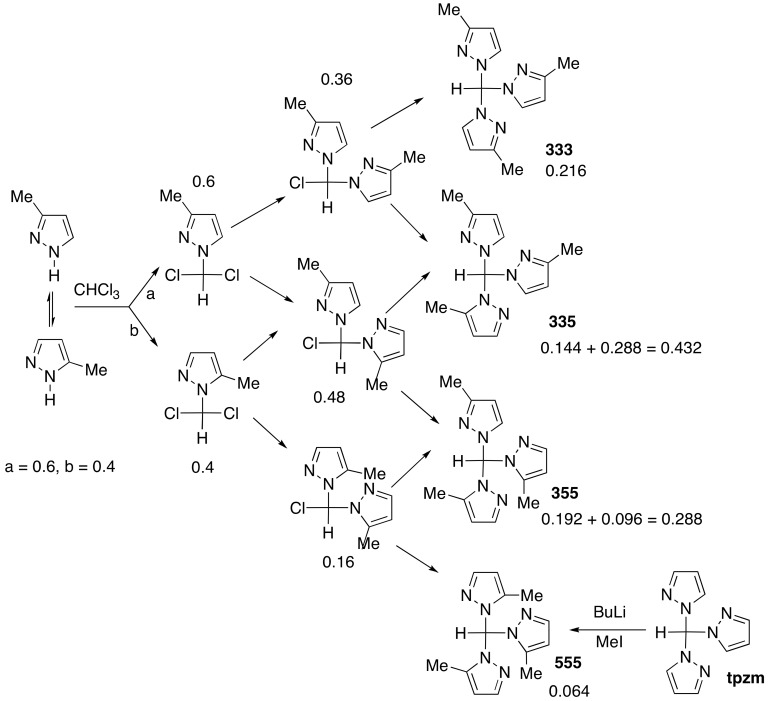
The four tris [3(5)-methyl]pyrazol-1-ylmethanes and the (0.6 + 0.4)^3^ proportions.

*N*-unsubstituted pyrazoles, and in general *N*-unsubstituted azoles, react with chloroform in phase-transfer catalysis (PTC) conditions to afford trispyrazolylmethanes [1], the neutral equivalents of anionic scorpionates [2]. The reaction of 3(5)-methyl-1*H*-pyrazole (**1**) with chloroform was reported in 1984 and the only compound isolated in a pure state was the tris(3-methylpyrazol-1-yl)methane **333** derivative (^1^H, CDCl_3_, Csp^3^-H: 8.11 ppm) (Figure 1) [3]. We have used a double nomenclature, 3 or 5 to define the position of the methyl group (3 precedes 5) and down (*d*) and up (*u*) to define the position of N2 (N4, N6) with regard to the H of the Csp^3^–H group (down on opposite sides, up on the same side). For instance, structure types **335** *duu* and **355** *duu* should be read 3Me-*d*, 3Me-*u*, 5Me-*u* and 3Me-*d*, 5Me-*u*, 5Me-*u*. We have used this nomenclature in previous papers [4,5,6].

The reaction was reported again in 1999 and, surprisingly, the only isolated isomer (17% yield) was the **335** isomer (^1^H, CDCl_3_, Csp^3^-H: 8.21 ppm) [7]. In 2012 the more hindered **555** derivative was prepared from tri(pyrazol-1-yl)methane (**tpzm**) by alkylation of the lithium derivative (^1^H, CDCl_3_, Csp^3^-H: 8.31 ppm) [8].

**Figure 2:**
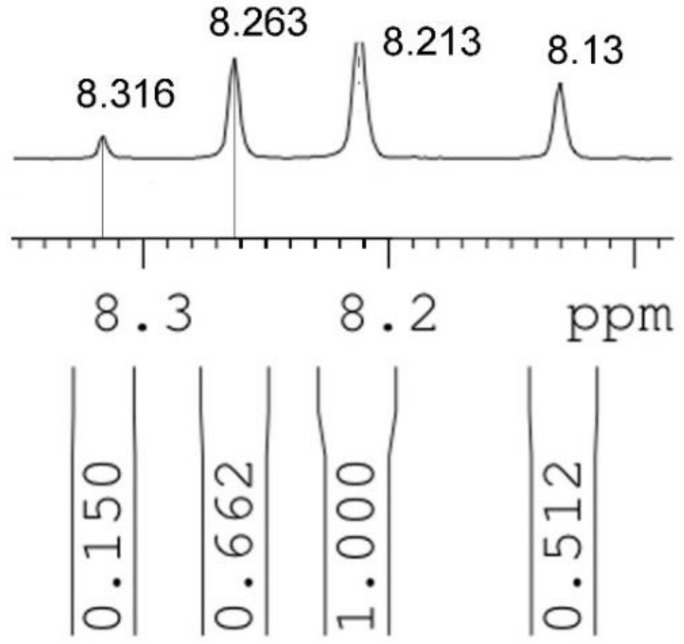
The ^1^H-NMR spectrum in CDCl_3_ of the Csp^3^-H proton of the crude mixture (integration in the vertical scale) [8].

Finally, the reaction was repeated again in 2012 and, although the yields of different isomers were not discussed, using the information concerning the ^1^H-NMR of the Csp^3^-H proton from this and others papers the relative yields of the four isomers can be determined (Figure 2) [8,9]. The authors also demonstrated that the mixture of the four isomers could be isomerized under the action of *p*-toluenesulfonic acid to a mixture of **333** and **335** in a 2:1 ratio (crude yield 82%), proving that they are the most stable isomers. The utility of the **333** ligand prompted Anwander et al. to prepare it from the mixture and isolate it by recrystallization [10].

The authors point out that the **333** isomer, *δ* = 8.13 ppm, represents approximately 22% of the crude. From the data of Figure 2 reported and from the integration (total 2.324) it is easy to determine the proportions: **333**, *δ* = 8.13 ppm, 22%; **335**, *δ* = 8.21 ppm, 43%; **355**, *δ* = 8.26 ppm, 28.5%; and **555**, *δ* = 8.32 ppm, 6.5%.

We have shown that in the reaction of polyhaloalkanes, such as dichloromethane, and chloroform with non-symmetrical 1*H*-pyrazoles (different substituents at position 3 and 5), the proportion of isomers in the crude always follows a binomial expansion, in this case (a + b)^3^, where a + b = 1 [11,12].

Figure 1 shows the results obtained with a = 0.6 and b = 0.4, i.e., more 3-methyl than 5-methyl isomers, a ratio consistent with the proportions obtained by alkylation of 3(5)-methyl-1*H*-pyrazole [13,14,15,16,17,18].

The calculated values (21.6%, 43.2%, 28.8% and 6.4%) are remarkably consistent, indicating that at every step the ratio 0.6/0.4 is constant. The accuracy is so remarkable that it is possible to conclude that a = 0.603, b = 0.397 yields better results: 21.9%, 28.5%, 43.3% and 6.3%.

**Figure 3:**
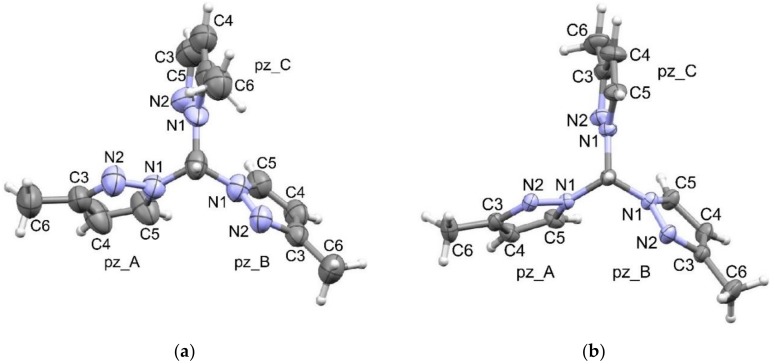
A view of the structure of (**a**) the **333** isomer (Cambridge Structural Database (CSD) [21] refcode: TUYZEU) and (**b**) the **335** isomer (bis(3-methylpyrazolyl, 5-methylpyrazolyl)methane). Displacement ellipsoids are drawn at 50% probability level. Hydrogen atoms are represented as spheres of 0.1Å radii.

The **333** compound prepared as in [3] was used to prepare complexes with Fe(II) to study their spin-transition temperatures [19]. The few relevant data concerning these compounds are ^13^C-NMR data (unassigned) of **555 [7]**, and the crystal structure of **333** (obtained by the general procedure followed by the isomerization of the mixture using *p*-toluenesulfonic acid) [20]. The structure of **333** (TUYZEU, Figure 3) [21] corresponds to a *uud* conformation. These isomerization experiments, together with those reported previously [9], prove that the stability decreases in the order **333** > **335** > **355** or **555**.

The melting points of three isomers were known: **333**, 107–109 °C [3], **335**, 114–115 °C [7], and **555**, 145–148 °C [8].

## 2. Results and Discussion

We decided to repeat the reaction shown in Figure 1 to determine if it was possible to isolate other isomers and establish their structures, and to discuss their conformations.

### 2.1. Chemistry

The synthesis of *N*,*N*’,*N*”-3(5)-trimethylpyrazolylmethanes was performed following the protocol described by Juliá et al. for the synthesis of *N*,*N*’,*N*”-triazolylmethanes (see Section 3.2) [3]. The percentages were determined by ^1^H-NMR using the signals of the Csp^3^-H atom in CDCl_3_ at 400 MHz (Table 1): 21.8% **333**, 47.8% **335**, 25.2% **355** and 5.2% **555**. These percentages correspond to (a + b)^3^ for a = 0.60 and b = 0.40 (21.6%; 43.2%; 28.8%; 6.4%) with a little worse agreement than the literature results [8].

### 2.2. NMR Studies

Table 1 (CDCl_3_) and Table 2 (C_6_D_6_) contain all the NMR information concerning the four isomers. The calculated values are in the Electronic Supplementary Information (ESI), Supplementary Materials Tables S1 to S4. In CDCl_3_ equilibration between isomers occurs that can be due to the presence of DCl, although we keep the solvent over Ag wire. For this reason, only the “fast” ^1^H-NMR experiments are reported in this solvent (see later). To avoid the problems encountered with deuterochloroform we move to another solvent. We select hexadeuterobenzene because in this solvent (in earlier work C_6_H_6_ was used) the methyl groups of pyrazoles have rather different ^1^H-NMR chemical shifts [22,24].

The methyl groups appear in C_6_D_6_ at ~2.10 (3-methyl) and ~1.85 ppm (5-methyl), very close to the values reported in the literature, 2.25 and 1.80 ppm, respectively [22,23,24]. These values together with the relative intensities allow an immediate identification of the four isomers by ^1^H-NMR.

Another useful criterion is that ^3^*J*_HH_ has a value of 1.8 Hz between H3 and H4 protons and 2.6 Hz between H3 and H4 protons. When a methyl group was involved, then ^4^*J*_H4Me5_ > ^4^*J*_H4Me3_ [22,23,24].

The GIAO calculated chemical shifts reported in the ESI agree well with the experimental values. To determine the major conformers, the problem is that the variation of chemical shift inter-conformers are small compared with the variation within each isomer that results in correlation coefficients R = 1.000 or 0.999 in most cases (correlation coefficient matrix). However, considering not only R^2^, but also an intercept as small as possible and a slope as close to 1.00 as possible, the results of Table 3 were obtained. 

They are not all consistent but clear preferences are observed. We have tried a mixture of the conformations of lower energy; for the **335** isomer we have preferred the *udd* (12.6 kJ·mol^–1^) to the *uuu* (12.1 kJ·mol^–1^).

**333** = −(0.2 ± 0.3) + (0.95 ± 0.16) *uud* + (0.06 ± 0.16) *uuu*, n = 20, R^2^ = 1.000, RMS = 0.8 ppm (1)

**335** = −(0.4 ± 0.2) + (0.30 ± 0.07) *udd* + (0.72 ± 0.07) *uud*, n = 20, R^2^ = 1.000, RMS = 0.8 ppm (2)

**355** = −(0.7 ± 0.5) + (0.75 ± 0.04) *ddu* + (0.25 ± 0.04) *duu*, n = 20, R^2^ = 1.000, RMS = 2.0 ppm (3)

In the case of the **555** isomers, the regression leads to a negative coefficient for the *uud* isomer that does not have a physical meaning, which indicates that the only isomer present is the *udd* isomer.

The mixtures (sum ≈ 1.00) correspond to about 3/4 of the lower energy isomers and 1/4 of the higher energy ones for **335** and **355**; in the case of the **333** isomer there is 95% of the *uud* isomer.

**Figure 4:**
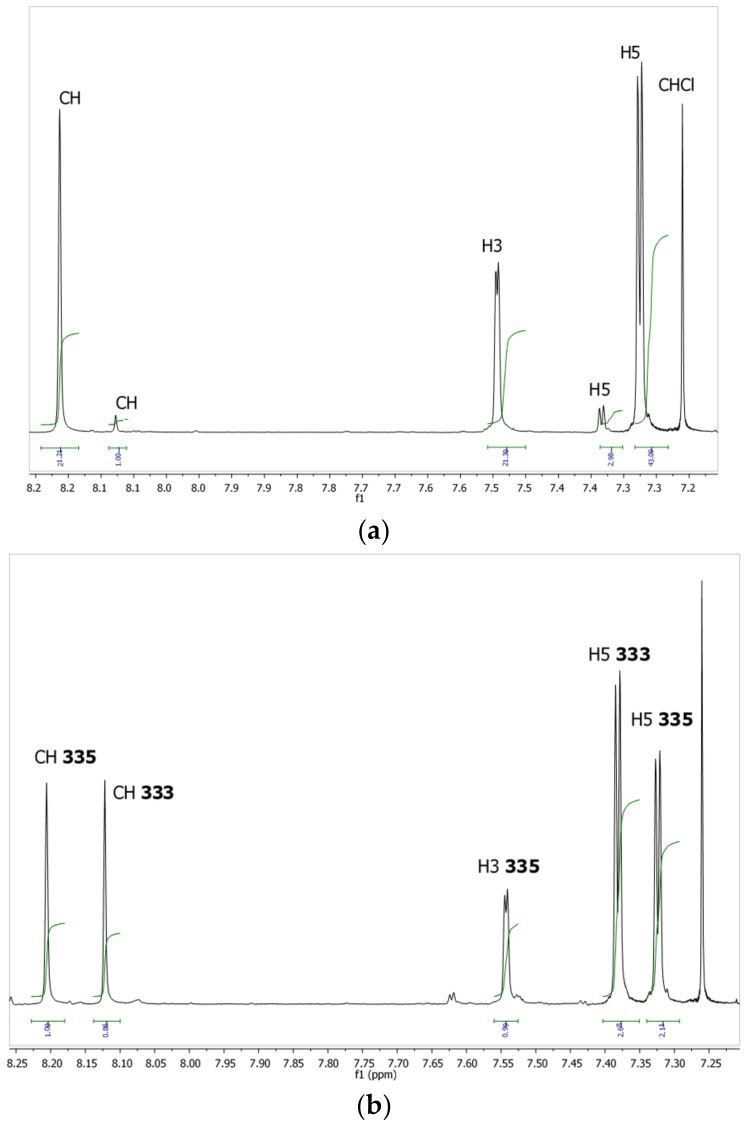
Evolution of the **333/335** ratio with time. (**a**) Recorded after 1 h in CDCl_3_, about 95% of **335** isomer and 5% of **333** isomer; (**b**) recorded after one night in CDCl_3_, about 50/50 of **335** and **333** isomer.

We found that a solution of an almost pure sample of the **335** isomer in CDCl_3_ slowly isomerizes into the more stable **333** isomer (Figure 4). This could be due to the presence of DCl in the solvent produced by photodecomposition of CDCl_3_, and is related to the already reported isomerization in acid media [9]. The mechanism should proceed by protonation of one of the pyrazoles (formation of a pyrazolium salt), leaving this ring as neutral 3(5)-methyl-1*H*-pyrazole, and the resulting carbocation reacting with 3(5)-methyl-1*H*-pyrazole.

### 2.3. Crystallography

The crystal structure of **335** presents one independent molecule in its asymmetric unit with two of their pyrazole N2 atoms pointing to the CH direction and the third one pointing into the opposite direction; therefore, these molecules present an *uud* conformation displaying HCN1N2 dihedral angles of 39.0(6)°, 17.6(7)° and −168.1(3)°. As the crystal space group contains inversion centers, both enantiomers coexist in a 1:1 ratio.

A summary of the crystal data and structure refinement is included in the ESI as Table S1. Table 4 contains geometrical parameters of 3(5)-methylpyrazolylmethane isomers, the one recorded in the Cambridge Structural Database (CSD) [21] and the new one reported in this manuscript. A view of their molecule structure with their atom labeling is depicted in Figure 3.

**Figure 5:**
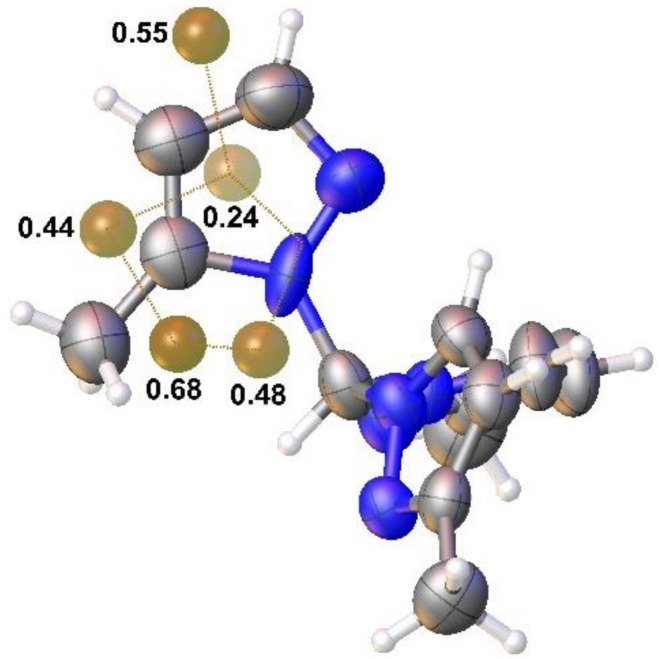
A view of the molecular structure of compound **335** showing the highest difference peaks (e Å^−3^). Dotted lines joining these peaks mimic the disorder model observed and refined for the other crystal collected at low temperature.

There are no significant differences in the observed geometry parameters for the pyrazole rings for the 3-methyl rings in both **333** and **335** isomers; however, the 5-methyl ring in **335** presents unusual geometries, especially for the intra-ring bond angles, N2-C3-C4, C3-C4-C5 and C4-C5-N1, and also for the N1-C5-C6 and C4-C5-C6 angles. There is not any apparent reason to justify this, but X-ray data collected for other crystals showed a possible occupancy disorder of the two isomers, **335** and **333**. Therefore, pz_C can, overall, be of 5-methylpyrazolyl with high occupancy and 3-methylpyrazolyl at low occupancy. It also justifies the difference peaks observed around the pz_C ring (Figure 5). A disorder model has been able to be refined with a crystal collected at low temperature but, due to the problems in reaching refinement convergence with the data, and the many geometrical restraints and constraints that were necessary, we do not think that it adds any knowledge to the results presented in this manuscript.

Both compounds present a twist of the pz-planes describing a propeller structure independently of the N2 position (up or down).

**Figure 6:**
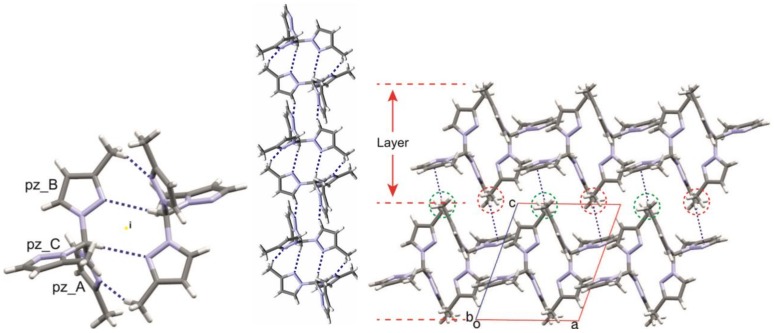
Three views of the structure of compound **335**.

Compound **335** forms dimers through weak CH···N hydrogen bonds (C1-H···N2(B), C6(B)-H···N2(A)) that expand into chains along (1–10) axis by C5(B)-H···N2(C) contacts. Saturating the three N-acceptors in the molecules, these chains join to form (001) layers by C-H···π-pz non-bonded interactions (C3(C)-H···π-pz(A), C4(C)-H···π-pz(B)). Methyl groups of pyrazoles A and B point out of these layers forming lines along the *b*-direction with an *a*-axis separation between methyl lines where a methyl line, from a consecutive layer, fits as a zipper to pack the layers and to build the crystal. C6(A)-H···π-pz(C) and van der Waal interactions glue the layers (Figure 6).

**Figure 7:**
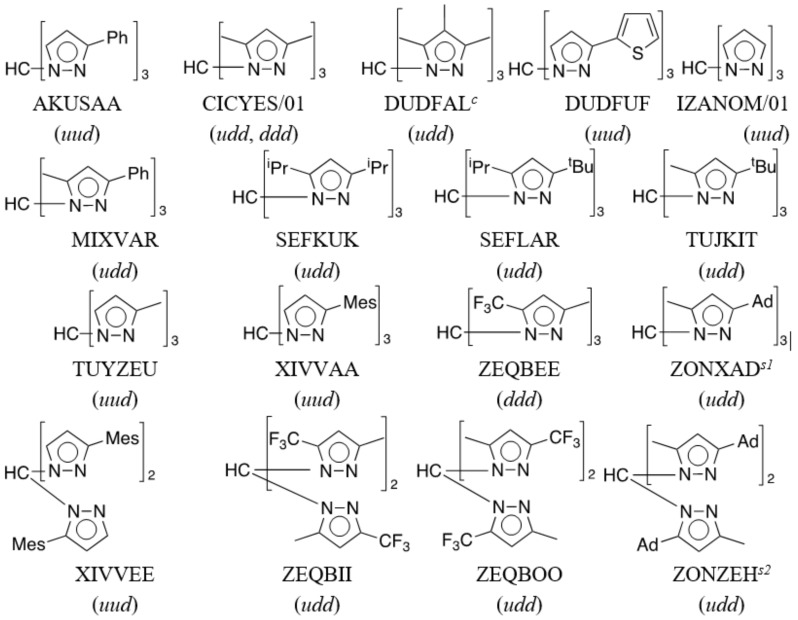
Molecular diagram of 17 trispyrazolylmethane derivatives recorded in the CSD version 5.39, updates to Feb 2018. (*^c^* cocrystallized with tmeda, *^s1^* benzene and *^s2^* n-hexane solvates).

In the Cambridge Structural Database (CSD, Version 5.39, updates to Feb 2018) [21], 19 structures of 17 trispyrazolylmethane compounds are recorded (Figure 7). Five of these structures contain 3(5)-pyrazoles, and the observed isomers are **333** in four cases (AKUSAA, DUDFUF, TUYZEU, XIVVAA) and **335** in the remaining one (XIVVEE). The one we report here is the second example of a **335** isomer.

All trispyrazolylmethane molecules display a propeller structure with a wide range of twisting in their pyrazole planes, from 3° to 68°. The most frequent conformations are *udd* (observed in 10 structures) and *uud* (observed in seven structures).

### 2.4. Theoretical Calculations

**Figure 8:**
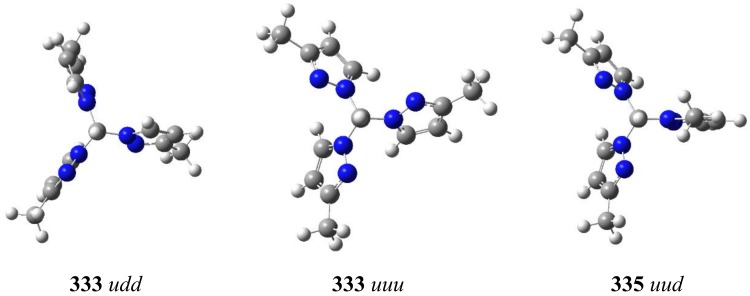

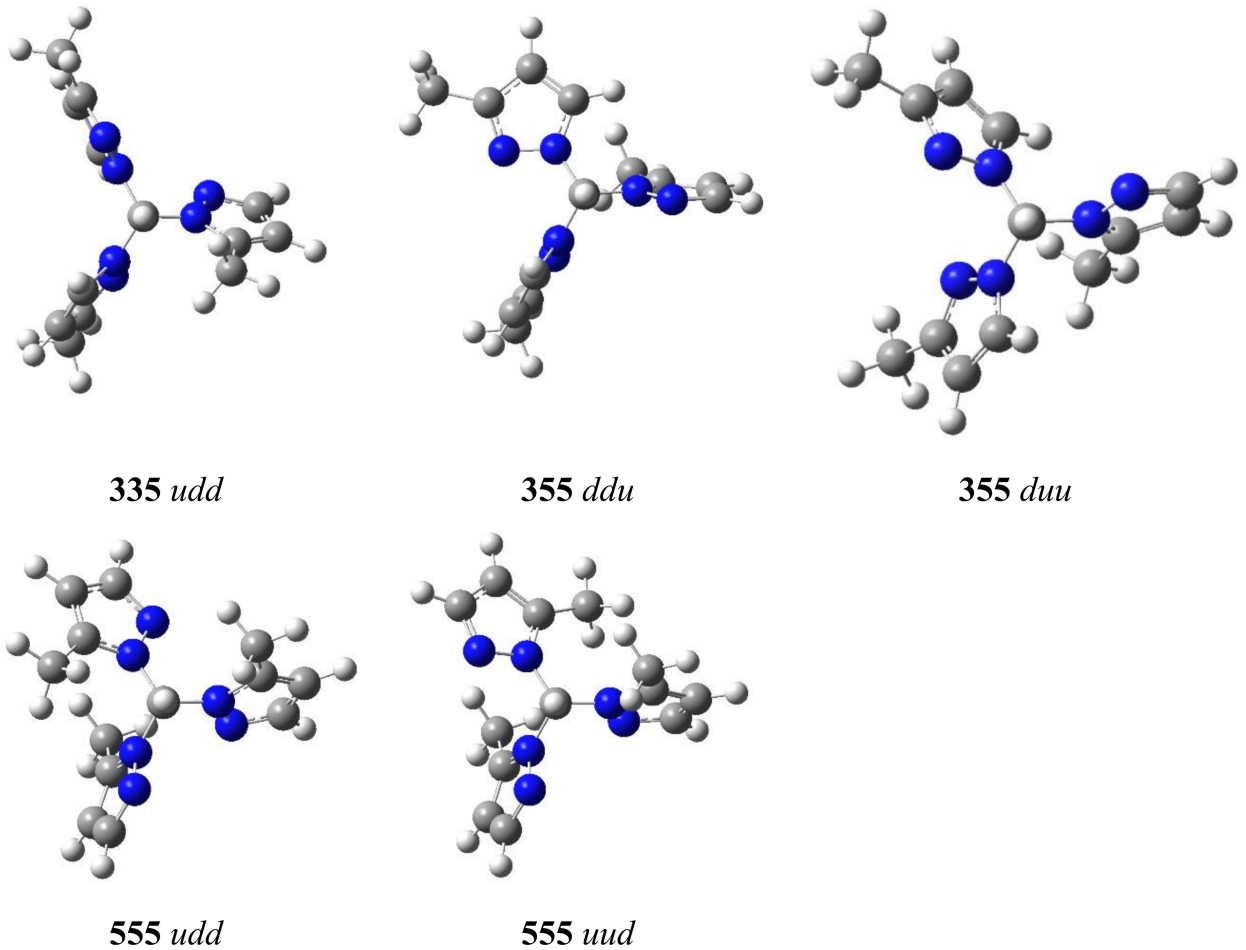
The most relevant calculated structures (the geometries of all the structures are to be found in the ESI).

The geometries of the two most relevant isomers are depicted in Figure 8 while the energies are reported in Table 5.

The isomers’ stability decreases in the order **333** (0.0) > **335** (1.7) > **355** (17.9) > **555** (23.8 kJ·mol^−1^), in agreement with the experimental results; this is the thermodynamic order that is unrelated to the kinetic order of the percentages measured on the crude. The acid-catalyzed isomerization from the crude leads to a mixture of **333** and **335** which have very close energies (Figure 7) [8]. Concerning the up/down isomerism, the most stable are the ***uud*** ones (**333**, **335**), the ***ddu*** one (**355**) and the ***udd*** one (**555**). The **355** *uud* is 9.9 kJ·mol^−1^ above the **355** *ddu* and the **555** *uud* is 5.9 kJ·mol^−1^ above the **555 *udd*** one.

Concerning the calculated geometries, the most interesting parameters are the torsion angles (Table 6).

The chemical shifts we have used for the interpolations (Equations (1–3)) have been obtained transforming the absolute shielding calculated with the B3LYP/6-311++G(d,p)/GIAO methods for the gas phase and then transformed through empirical equations to chemical shifts (see Section 3.5). These *δ* values do not correspond to the gas phase but to solution, because the empirical equations were established using gas phase *σ* and solution *δ*.

When comparing the experimental chemical shifts in solution to the GIAO calculated ones, remember that the four **3/5** isomers correspond to different molecules that are stable in the NMR time scale. On the other hand, the up/down rotational isomers are separated by low rotational barriers and in solution only averaged signals will be observed.

## 3. Materials and Methods

### 3.1. Experimental

High-resolution mass spectra were recorded on a Quadrupole Time-of-Flight (QTOF) mass spectrometer under Electrospray Ionization (ESI) conditions.

### 3.2. Chemistry

A mixture of 3(5)-methyl-1*H*-pyrazole (1.93 mL, 24 mmol), anhydrous K_2_CO_3_ (16.58 g, 120 mmol) and (Bu)_4_NHSO_4_ (0.41 g, 1.2 mmol) was vigorously stirred and refluxed in dry CHCl_3_ (25 mL) for 24 h. Then the mixture was filtered and the residue washed with hot CHCl_3_ (3 × 25 mL). The organic solution was evaporated and the crude product was purified by column chromatography (using silica 1:130) and then by crystallization using a mixture of diethyl ether/hexane. Four isomers of *N*,*N*’,*N*”-3(5)-trimethylpyrazolylmethanes were formed: the 3,3,3-trimethylpyrazolylmethane (**333**), the 3,3,5-trimethylpyrazolylmethane (**335**), which was the major isomer, the 3,5,5-trimethylpyrazolylmethane (**355**) and the 5,5,5-trimethylpyrazolylmethane (**555**), which was the minor isomer. The overall reaction yield calculated as the sum of all the isomers isolated by column chromatography was 46% (0.94 g), corresponding to 0.20 g of **333**, 0.45 g of **335**, 0.24 g of **355** and 0.05 g of **555**. Melting points were determined under microscope.

Only the **335** isomer was isolated pure in enough quantity; the **355** was isolated in a very small amount only enough to measure its melting point and record its exact mass; the two other isomers, **333** and **555** only as mixtures of two isomers enriched in one of them. 

Tris(3-methyl-1*H*-pyrazol-1-yl)methane (**333**): Not isolated pure. According to literature it melts at 107–109 °C [3].

Bis(3-methyl-1*H*-pyrazol-1-yl)(5-methyl-1*H*-pyrazol-1-yl)methane (**335**): M.p. = 109–111 °C, literature: 114–115 °C [7]. HRMS (ESI) [M + H]^+^ Calcd for [C_13_H_17_N_6_^+^] 257.1509, found 257.1506.

Bis(5-methyl-1*H*-pyrazol-1-yl)(3-methyl-1*H*-pyrazol-1-yl)methane (**355**): M.p. = 55–57 °C. HRMS (ESI) [M + H]^+^ Calcd for [C_13_H_17_N_6_^+^] 257.1509, found 257.1512.

Tris(5-methyl-1*H*-pyrazol-1-yl)methane (**555**): Not isolated pure. According to literature it melts at 145–148 °C [8].

### 3.3. NMR Spectroscopy

Solution NMR spectra were recorded on a 9.4 Tesla Bruker spectrometer (Bruker Española S.A., Madrid, Spain), 400.13 MHz for ^1^H, 100.62 MHz for ^13^C and 40.54 MHz for ^15^N) at 300 K with a 5-mm inverse detection H-X probe equipped with a z-gradient coil. Chemical shifts (*δ* in ppm) are given from internal solvents: CDCl_3_ 7.26 for ^1^H; C_6_D_6_ 7.16 for ^1^H and 128.39 for ^13^C. Nitromethane was used as external reference for ^15^N. Coupling constants (*J* in Hz) are accurate to ±0.2 Hz for ^1^H and ±0.6 Hz for ^13^C. CDCl_3_ contains 0.5 wt% silver wire as stabilizer.

Typical parameters for ^1^H-NMR spectra were spectral width 4000 Hz and pulse width 9.5 μs at an attenuation level of 0 dB. Typical parameters for ^13^C-NMR spectra were spectral width 21 kHz, pulse width 10.6 μs at an attenuation level of −6 dB and relaxation delay 2 s. WALTZ 16 was used for broadband proton decoupling; the FIDs were multiplied by an exponential weighting (lb = 2 Hz) before Fourier transformation. In some cases, for resolution enhancement processing a Gaussian multiplication of the FID prior to Fourier transformation was applied.

2D (^1^H-^13^C) gs-HMQC, (^1^H-^13^C) gs-HMBC and (^1^H-^15^N) gs-HMBC, were acquired and processed using Bruker NMR software suite (Bruker, Karlsruhe, Germany) in non-phase-sensitive mode. Gradient selection was achieved through a 5% sine truncated shaped pulse gradient of 1 ms. 

Selected parameters for (^1^H-^13^C) gs-HMQC and gs-HMBC spectra were: spectral width 4000 Hz for ^1^H and 20 kHz for ^13^C, 1024 × 256 data set, number of scans 2 (HMQC) or 4 (HMBC) and relaxation delay 1s. In the gs-HMQC experiments GARP modulation of ^13^C was used for decoupling. The FIDs were processed using zero filling in the *F*1 domain and a sine-bell window function in both dimensions was applied prior to Fourier transformation.

Selected parameters for gs-HMBC spectra were: spectral width 4000 Hz for ^1^H and 15 kHz for ^15^N, 2048 × 1024 data set, number of scans 4, relaxation delay 1s. In the gs-HMBC delays of 60 and 100 ms for the evolution of the ^15^N-^1^H long-range coupling were used. The FIDs were processed using zero filling in the *F*1 domain and a sine-bell window function in both dimensions was applied prior to Fourier transformation.

### 3.4. Crystallography

For compound **335** [bis(3-methylpyrazolyl)(5-methylpyrazolyl)methane], X-ray single crystal diffraction data were collected on a Bruker APEX-II CCD diffractometer (Bruker Española S.A., Madrid, Spain) [25]. 

From initial data collected at room temperature, a possible occupancy disorder of the two isomers, **335** and **333** was identified. Data collected at 150 K of a different crystal allowed us to build a disorder model that showed a mixture of these two isomers. Due to the many problems to refine this disordered model and to reach convergence, we kept the best data for the work presented in this manuscript. It corresponds to data collected at room temperature and it shows the lowest proportion of the **333** isomer (so it could be omitted).

Using Olex2 (v1.2, Durham University, Durham, UK) [26], the structure was solved with the ShelXS (v4-2016, Universität Göttingen, Göttingen, Germany) [27] structure solution program using Direct Methods and refined with the ShelXL (v4-2016, Universität Göttingen, Göttingen, Germany) [28] refinement package using Least Squares minimization. A summary of the crystal data and structure refinement is included in Table S5. For the visualization and analysis of crystal structures the Mercury program was used [29].

### 3.5. Theoretical Calculations

Density Functional Theory (DFT) calculations were carried out using the Becke, three-Parameter, Lee, Yang and Parr (B3LYP) Gaussian 09 (Version D.01, Wallingford, CT, USA, 2009) [30,31,32], together with the 6-311++G/(d,p) basis set [33,34]. Absolute shieldings were calculated within the GIAO approximation [35,36]. All the calculations were carried out using the Gaussian 09 package (Version D.01, Wallingford, CT, USA, 2009) [37]. Empirical equations were used to transform the ^1^H, ^13^C and ^15^N absolute shieldings into chemical shifts [38,39].

## 4. Conclusions

The accuracy of the polynomial expansion (a + b)^3^ is extraordinary because it was unexpected. It implies that the ratios of the reactivity of the chlorine atoms with 3(5)-methyl-1*H*-pyrazole are the same for CHCl_3_, CH(Mepz)Cl_2_ and CH(Mepz)_2_Cl. This work reported the first systematic study of the structure of the four tris[3(5)-methyl]pyrazol-1-ylmethanes, a series of ligands used in coordination chemistry.

The solid-solution [40] structure of the **335** isomer, actually a mixture of **335** (major) and **333** (minor) isomers, results from the isomerization of the **335** isomer into the **333** isomer during the crystallization process. In the crystallization batch there are different crystals but a clear predominance of the **335** isomer is always found by crystallography.

## Figures and Tables

**Table 1:** ^1^H-NMR data of trispyrazolylmethanes at 400.13 MHz. Solvent CDCl_3_. Chemical shifts (*δ*, ppm) in ppm; ^1^H-^1^H spin-spin coupling constants *J* (Hz).

Isomer	3-Methyl	5-Methyl	CH
**333**	H4: 6.11 (d, ^3^*J*_H4H5_ = 2.5) H5: 7.38 (d, ^3^*J*_H5H4_ = 2.5) CH_3_: 2.27 (s)	----	8.12 8.11 [8]
**335**	H4: 6.11 (d, ^3^*J*_H4H5_ = 2.5) H5: 7.33 (d, ^3^*J*_H5H4_ = 2.5) CH_3_: 2.27 (s)	H3: 7.55 (d, ^3^*J*_H3H4_ = 1.5) H4: 6.11 (dq, ^3^*J*_H4H3_ = 1.5, ^4^*J*_H4Me_ = 0.4) CH_3_: 2.39 (d, ^4^*J*_MeH4_ = 0.4)	8.21 8.21 [8]
**355**	H4: 6.12 (dq, ^3^*J*_H4H5_ = 2.5, ^4^*J*_MeH4_ = 0.4) H5: 7.33 (d, ^3^*J*_H5H4_ = 2.5) CH_3_: 2.29 (d)	H3: 7.37 (dq,^3^*J*_H3H4_ = 1.7, ^5^*J*_H3Me_ = 0.4) H4: 5.72 (dq, ^3^*J*_H4H3_ = 1.7, ^4^*J*_H4Me_ = 0.8) CH_3_: 2.23 (dd, ^4^*J*_MeH4_ = 0.8, ^5^*J*_MeH3_ = 0.4)	8.26 (s) 8.26 [8]
**555**	----	H3: 7.51 (ddq, ^3^*J*_H3H4_ = 1.6, ^5^*J*_MeH3_ = 0.4) H4: 5.80 (dq, ^3^*J*_H4H3_ = 1.7, ^4^*J*_H4Me_ = 0.8) CH_3_: 2.09 (dd, ^4^*J*_MeH4_ = 0.8, ^5^*J*_MeH3_ = 0.4)	8.31 (s) 8.33 [8]

**Table 2:** ^1^H (400.13 MHz), ^13^C (100.62 MHz) and ^15^N (40.54 MHz) NMR data of trispyrazolylmethanes. Solvent C_6_D_6_. Chemical shifts (*δ*, ppm) in ppm; ^1^H-^1^H and ^1^H-^13^C spin-spin coupling constants *J* (Hz).

Isomer	3-Methyl	5-Methyl	CH
**333**	N1: –173.3 N2: –79.5	----	----
	C3: 150.9 C4: 107.1, ^1^*J* = 175.3, ^2^*J* = 6.8, ^3^*J* = 3.4 C5: 130.6, ^1^*J* = 188.5, ^2^*J* = 9.5, ^3^*J* = 2.5 CH_3_: 14.0, ^1^*J* = 127.3	----	83.8 ^1^*J* = 166.4
	H4: 5.76 (d, ^3^*J*_H4H5_ = 2.5) H5: 7.27 (d, ^3^*J*_H5H4_ = 2.5) CH_3_: 2.08 (s)	----	8.22
**335**	N1: –172.8 N2: –77.9	N1: –171.4 N2: –80.4	----
	C3: 150.5 C4: 107.3, ^1^*J* = 175.0, ^2^*J* = 8.3, ^3^*J* = 3.3 C5: 130.3, ^1^*J* = 189.3, ^2^*J* = 9.5, ^3^*J* = 2.6 CH_3_: 13.9, ^1^*J* = 127.2	C3: 141.2, ^1^*J* = 185.2, ^2^*J* = 5.7 C4: 107.0, ^1^*J* = 175.1, ^2^*J* = 10.6, ^3^*J* = 3.8 C5: 140.3 CH_3_: 10.5, ^1^*J* = 128.5	81.4 ^1^*J* = 164.2
	H4: 5.82 (d, ^3^*J*_H4H5_ = 2.5) H5: 7.56 (d, ^3^*J*_H5H4_ = 2.5) CH_3_: 2.11 (s)	H3: 7.36 (d, ^3^*J*_H3H4_ = 1.7) H4: 5.62 (dq, ^3^*J*_H4H3_ = 1.7, ^4^*J*_H4Me_ = 0.8) CH_3_: 1.82 (d, ^4^*J*_MeH4_ = 0.8)	8.40 (s)
**355**	N1: –175.6 N2: –78.8	N1: –171.3 N2: –76.5	----
	C3: 151.0 C4: 106.8, ^1^*J* = 175.3 C5: 131.3,^1^*J* = 190.3, ^2^*J* = 9.9 CH_3_: 14.0, ^1^*J* = 127.3	C3: 140.7, ^1^*J* = 185.1, ^2^*J* = 5.8 C4: 107.8, ^1^*J* = 174.8 C5: 140.5 CH_3_: 10.9, ^1^*J* = 129.1	81.6 ^1^*J* = 164.6
	H4: 5.82 (^3^*J*_H4H5_ = 2.5) H5: 7.28 (^3^*J*_H5H4_ = 2.5) CH_3_: 2.14(s)	H3: 7.37 (^3^*J*_H3H4_ = 1.7) H4: 5.72 (dq, ^3^*J*_H4H3_ = 1.7, ^4^*J*_H4Me_ = 0.8) CH_3_: 1.87 (m)	8.46 (s)
**555**	----	N1: –173.4 N2: –75.4	----
	----	C3: 140.1, ^1^*J* = 185.0, *J* = 5.8 C4: 108.1, ^1^*J* = 174.7 (m) C5: 140.5 CH_3_: 10.9, ^1^*J* = 128.6	82.1 ^1^*J* = 163.1
	----	H3: 7.37 (^3^*J*_H3H4_ = 1.7, H3) H4: 5.80 (dq, ^3^*J*_H4H3_ = 1.7, ^4^*J*_H4Me_ = 0.8) CH_3_: 1.84 (m)	8.43 (s)

**Table 3:** Best conformers according to the method. ^1^H in CDCl_3_; ^13^C and ^15^N in C_6_D_6._

Comp	Conformation	E_rel_	^1^H	^13^C	^15^N	X-ray
**333**	*ddd*	37.1	*ddd*			
	*udd*	11.2			*udd*	
	*uud*	**0.0**	*uud*	*uud*	*uud*	*uud:* TUYZEU
	*uuu*	2.4		*uuu*		
**335**	*ddd*	37.4				
	*ddu*	15.1	*ddu*			
	*duu*	17.3	*duu*			
	*udd*	12.6		*udd*	*udd*	
	*uud*	**0.0**		*uud*	*uud*	*uud:* this work
	*uuu*	12.1				
**355**	*ddd*	23.2				
	*ddu*	**0.0**	*ddu*		*udd*	
	*duu*	2.5	*duu*			
	*udd*	4.1		*udd*		
	*uud*	9.9				
	*uuu*	10.7		*uuu*		
**555**	*ddd*	19.3				
	*udd*	**0.0**	*udd*	*udd*	*udd*	
	*uud*	5.9	*uud*	*uud*	*uud*	
	*uuu*	18.5				

**Table 4:** Selected geometrical parameters for **333** and **335.**

	333*uud* (TUYZEU)			335*uud*		
	pz_A	pz_B	pz_C	pz_A	pz_B	pz_C
N1-N2	1.359	1.362	1.355	1.359(5)	1.364(5)	1.346(6)
N2-C3	1.330	1.328	1.335	1.328(6)	1.332(5)	1.351(7)
C3-C4	1.401	1.402	1.395	1.375(7)	1.383(6)	1.338(8)
C4-C5	1.361	1.362	1.357	1.360(7)	1.343(6)	1.339(7)
N1-C5	1.349	1.351	1.347	1.326(5)	1.350(5)	1.402(6)
C1-N1	1.445	1.444	1.440	1.453(5)	1.455(5)	1.483(6)
C5-N1-N2	111.8	112.1	112.5	111.2(4)	111.8(3)	112.0(4)
N1-N2-C3	104.8	104.6	104.3	105.1(4)	104.1(3)	105.0(4)
N2-C3-C4	110.9	111.0	110.7	110.5(4)	110.9(4)	108.8(5)
C3-C4-C5	105.5	105.8	106.2	106.1(4)	106.9(4)	112.1(5)
C4-C5-N1	107.0	106.5	106.2	107.2(4)	106.4(4)	102.1(5)
C1-N1-N2	117.5	117.4	121.5	117.0(3)	118.3(3)	119.6(3)
N2/1-C3/5-C6	120.2	120.4	120.6	120.8(5)	120.5(4)	120.5(5)
C4-C3/5-C6	128.9	128.5	128.7	128.6(5)	128.7(4)	137.5(5)
H1-C1-N1-N2	24.5	28.0	−170.9	39.0(6)	17.6(7)	−168.1(3)
H1-C1-N1-C5	−164.8	−160.0	10.8	−150.3(5)	−163.4(5)	15.3(5)
C1-N1-N2-C3	173.4	173.6	−178.4	172.6(4)	179.9(4)	−177.7(4)

**Table 5:** Energies (kJ·mol^–1^). The minimum energy conformation in black; the crystallographic structure, TUYZEU, in italics.

Compound	Conformation	Relative Energy, Conformations	Relative Energy, Isomers
**333**	*ddd*	37.1	
	*udd*	11.2	
	*uud*	**0.0**	**0.0**
	*uuu*	2.4	
TUYZEU	*uud*	*0.0*	
**335**	*ddd*	37.4	39.1
	*ddu*	15.1	16.8
	*duu*	17.3	19.0
	*udd*	12.6	14.2
	*uud*	**0.0**	**1.7**
	*uuu*	12.1	13.8
**355**	*ddd*	23.2	41.0
	*ddu*	**0.0**	**17.9**
	*duu*	2.5	20.4
	*udd*	4.1	22.0
	*uud*	9.9	27.8
	*uuu*	10.7	28.6
**555**	*ddd*	19.3	43.1
	*udd*	**0.0**	**23.8**
	*uud*	5.9	29.7
	*uuu*	18.5	42.3

**Table 6:** Torsions (°). The minimum energy conformation in black; the crystallographic structure, TUYZEU, in italics.

Compound	Conformation	8-7-1-23	13-12-1-23	3-2-1-23
**333**	*ddd*	145.3	145.3	145.3
	*udd*	**−5.5**	**177.7**	**159.1**
	*uud*	−32.9	−28.1	174.4
	*uuu*	−40.5	−40.5	−40.5
TUYZEU	*uud*	−32.9	−28.2	174.4
X-ray	*uud*	−27.97	−24.48	170.87
**335**	*ddd*	−143.4	−149.9	−136.1
	*ddu*	−173.0	−85.7	12.3
	*duu*	145.3	−38.6	−26.9
	*udd*	−170.1	4.7	−151.7
	*uud*	**−31.7**	**−26.7**	**174.9**
	*uuu*	−52.1	−41.1	−29.0
X-ray	*uud*	−39.0(6)	−17.4(7)	168.2(3)
**355**	*ddd*	−136.2	−147.6	−139.6
	*ddu*	**−173.0**	**−85.7**	**12.3**
	*duu*	145.3	−38.6	−26.9
	*udd*	−170.1	4.7	−151.7
	*uud*	−57.9	162.3	−22.9
	*uuu*	−43.3	−43.1	−34.4
**555**	*ddd*	139.2	139.2	139.2
	*udd*	**−18.4**	**129.3**	**151.6**
	*uud*	−24.2	−50.0	156.1
	*uuu*	−40.2	−40.2	−40.2

## Supplementary Materials

Electronic supplementary information (ESI) is available online at http://www.mdpi.com/1420-3049/24/3/568/s1: calculated NMR chemical shifts (GIAO), theoretical calculations (Tables S1 to S4), Crystallographic details (Table S5), CCDC 1875403 for compound **335**.
