# Supplementary material for: An Example of Polynomial Expansion: The Reaction of 3(5)-Methyl-1H-Pyrazole with Chloroform and Characterization of the Four Isomers
Source: Molecules. 2019 Feb 4;24(3):568. doi: 10.3390/molecules24030568 (PMC6384863; doi:10.3390/molecules24030568)
Supplement: Supplementary file 1 [file molecules-24-00568-s001.zip › ESI_NMR_GIAO.docx]

**Electronic Supporting Information**

An example of polynomial expansion: the reaction of 3(5)-methyl-1*H*-pyrazole with chloroform and characterization of the four isomers†

Vera L. M. Silva ^1^, Artur M. S. Silva ^1,^*, Rosa M. Claramunt ^2^, Dionisia Sanz ^2^, Lourdes Infantes ^3,^*, Ángela Martínez-López ^3^, Felipe Reviriego ^4^, Ibon Alkorta ^4^ and José Elguero ^4^

^1^ Chemistry Department and QOPNA and LAQV-REQUIMTE, University of Aveiro, 3810-193 Aveiro, Portugal; [verasilva@ua.pt](mailto:verasilva@ua.pt) and artur.silva@ua.pt

^2^ Departamento de Química Orgánica y Bio-Orgánica, Facultad de Ciencias, UNED, Paseo Senda del Rey, 9, E-28040 Madrid, Spain; [rclaramunt@ccia.uned.es](mailto:rclaramunt@ccia.uned.es) and dsanz@ccia.uned.es

^3^ Departamento de Cristalografía y Biología Estructural, Instituto de Química-Física Rocasolano, CSIC, Serrano, 119, E-28006 Madrid, Spain; [xlourdes@iqfr.csic.es](mailto:xlourdes@iqfr.csic.es) and [angela_villalba9@hotmail.com](mailto:angela_villalba9@hotmail.com)

^4^ Instituto de Química Médica, CSIC, Juan de la Cierva, 3, E-28006 Madrid, Spain; [freviriegop@ictp.csic.es](mailto:freviriegop@ictp.csic.es); [ibon@iqm.csic.es](mailto:ibon@iqm.csic.es) and jelguero@iqm.csic.es

***** Correspondence: [artur.silva@ua.pt](mailto:artur.silva@ua.pt) and [xlourdes@iqfr.csic.es](mailto:xlourdes@iqfr.csic.es) ; Tel.: +351-234-370714 and +34-91-5619400

**Table of contents**

| **Table ES1:** GIAO calculated ^1^H chemical shifts (ppm)………………………………………… | 3 |
| --- | --- |
| **Table ES2:** GIAO calculated ^13^C chemical shifts (ppm)………………………………………... | 4 |
| **Table ES3:** GIAO calculated ^15^N chemical shifts (ppm)………………………………………... | 5 |
| **Table ES4:** Free-Wilson matrix for GIAO calculated ^15^N chemical shifts……………………… | 6 |
| **Table ES5** Crystal data, data collection and structure refinement for compound **335**…………... | 7 |

| **Table ES1:** GIAO calculated ^1^H chemical shifts (ppm) | | | |
| --- | --- | --- | --- |
| Comp. | 3-Mepz | 5-Mepz | CH |
| **333** *ddd* | *d*: 2.16 (Me), 6.10 (H4), 7.22 (H5) | ---- | 7.44 |
| **333** *udd* | *u*: 2.17 (Me), 5.98 (H4), 8.59 (H5)  *d*: 2.20 (Me), 5.88 (H4), 7.20 (H5)  *d*: 2.26 (Me), 5.98 (H4), 7.28 (H5) | ---- | 7.28 |
| **333 *uud*** | ***u*: 2.17 (Me), 5.86 H4), 7.20 (H5)**  ***u*: 2.17 (Me), 6.06 (H4), 8.38 (H5)**  ***d*: 2.22 (Me), 5.97 (H4), 7.42 (H5)** | **----** | **7.57** |
| **333** *uuu* | *u*: 2.26 (Me), 5.97 (H4), 6.58 (H5) | ---- | 7.94 |
| **335**  *ddd* | *d*: 2.19 (Me), 6.16 (H4), 7.41 (H5)  *d*: 2.16 (Me), 6.10 (H4), 7.22 (H5) | ---- | 7.47 |
|  | ---- | *d*: 1.98 (Me), 5.94 (H4), 7.16 (H3) |  |
| **335**  *ddu* | *d*: 2.25 (Me), 6.02 (H4), 7.25 (H5)  *d*: 2.17 (Me), 6.08 (H4), 6.95 (H5) | ---- | 7.47 |
|  | ---- | *u*: 2.72 (Me), 6.02 (H4), 7.30 (H3) |  |
| **335**  *duu* | *u*: 2.21 (Me), 5.96 (H4), 7.31 (H5)  *d*: 2.15 (Me), 6.12 (H4), 7.37 (H5) | ---- | 7.98 |
|  | ---- | *u*: 1.91 (Me), 5.92 (H4), 7.31 (H3) |  |
| **335**  *udd* | *u*: 2.18 (Me), 5.96 (H4), 8.34 (H5)  *d*: 2.20 (Me), 5.96 (H4), 7.35 (H5) | ---- | 7.47 |
|  | ---- | *d*: 2.24 (Me), 5.92 (H4), 7.32 (H3) |  |
| **335**  ***uud*** | ***u*: 2.25 (Me), 6.07 (H4), 8.44 (H5)**  ***u*: 2.17 (Me), 6.88 (H4), 7.27 (H5)** | **----** | **7.83** |
|  |  | ***d*: 2.43 (Me), 5.86 (H4), 7.31 (H3)** |  |
| **335**  *uuu* | *u*: 2.26 (Me), 6.07 (H4), 8.44 (H5)  *u*: 2.17 (Me), 6.88 (H4), 7.27 (H5) |  | 8.04 |
|  |  | *u*: 1.57 (Me), 5.94 (H4), 7.36 (H3) |  |
| **355**  *ddd* | *d*: 2.16 (Me), 6.17 (H4), 7.23 (H5) | ---- | 7.51 |
|  | ---- | *d*: 2.06 (Me), 6.00 (H4), 7.17 (H3)  *d*: 1.99 (Me), 5.98 (H4), 7.23 (H3) |  |
| **355**  ***ddu*** | ***d*: 2.25 (Me), 6.07 (H4), 7.25 (H5)** | **----** | **7.62** |
|  | **----** | ***u*:** **2.09 (Me), 5.90 (H4), 7.25 (H3)**  ***d*: 1.89 (Me), 6.03 (H4), 7.20 (H3)** |  |
| **355**  *duu* | *d*: 2.26 (Me), 6.04 (H4), 7.39 (H5) |  | 7.84 |
|  |  | *u*: 2.01 (Me), 5.85 (H4), 7.18 (H3)  *u*: 1.77 (Me), 5.81 (H4), 7.36 (H3) |  |
| **355**  *udd* | *u*: 2.21 (Me), 6.01 (H4), 8.05 (H5) | ---- | 7.74 |
|  | ---- | *d*: 2.34 (Me), 5.85 (H4), 7.20 (H3)  *d*: 2.22 (Me), 5.93 (H4), 7.38 (H3) |  |
| **355** *uud* | *u*: 2.22 (Me), 6.00 (H4), 7.03 (H5) | ---- | 8.05 |
|  | ---- | *u*: 1.92 (Me), 5.90 (H4), 7.31 (H3)  *d*: 2.24 (Me), 5.98 (H4), 7.21 (H3) |  |
| **355**  *uuu* | *u*: 2.30 (Me), 6.00 (H4), 6.50 (H5) | ---- | 8.24 |
|  | ---- | *u*: 1.64 (Me), 5.85 (H4), 7.42 (H3)  *u*: 1.58 (Me), 5.90 (H4), 7.35 (H3) |  |
| **555**  *ddd* | ---- | *d*: 1.99 (Me), 6.02 (H4), 7.26 (H3) | 7.57 |
| **555**  ***udd*** | **----** | *u*: 2.07 (Me), 5.90 (H4), 7.25 (H3)  *d*: 1.97, 2.18 (Me), 6.01, 6.02 (H4), 7.14, 7.33 (H3) | **7.75** |
| **555** *uud* | ---- | *u*: 1.70, 1.90 (Me), 5.84, 5.84 (H4), 7.38, 7.19 (H3)  *d*: 2.32 (Me), 6.01 (H4), 7.35 (H3) | 8.08 |
| **555**  *uuu* | ---- | *u*: 1.52 (Me), 5.82 (H4), 7.39 (H3) | 8.40 |

| **Table ES2:** GIAO calculated ^13^C chemical shifts (ppm) | | | | | | | | |
| --- | --- | --- | --- | --- | --- | --- | --- | --- |
| Comp. | *u/d* | Position | Csp^3^ | C3 | C4 | C5 | Me3 | Me5 |
| **333** | *ddd* | 3Me-*d* | 82.4 | 149.3 | 107.2 | 127.5 | 13.9 | ---- |
|  | *udd* | 3Me-*u* | 82.8 | 148.6 | 105.5 | 133.7 | 13.9 | ---- |
|  |  | 3Me-*d* |  | 150.0  152.0 | 106.0  104.6 | 127.6  128.2 | 14.0  14.0 | ---- |
|  | ***uud*** | **3Me-*u*** | **83.8** | **152.0**  **150.3** | **106.0**  **105.3** | **130.4**  **127.8** | **13.6**  **13.7** | ---- |
|  |  | **3Me-*d*** |  | **152.0** | **104.8** | **130.4** | **14.0** |  |
|  | *uuu* | 3Me-*u* | 84.9 | 151.7 | 105.5 | 128.1 | 13.8 | ---- |
| **335** | *ddd* | 3Me-*d*  3Me-*d* | 81.3 | 149.3  150.1 | 107.6  107.0 | 127.7  128.2 | 13.9  14.0 |  |
|  |  | 5Me-*d* |  | 137.9 | 106.8 | 138.3 | ---- | 10.6 |
|  | *ddu* | 3Me-*d*  3Me-*d* | 86.4 | 151.9  150.8 | 104.7  107.2 | 133.0  129.2 | 14.1  14.0 | ---- |
|  |  | 5Me-*u* |  | 139.1 | 109.0 | 143.7 | ---- | 13.3 |
|  | *duu* | 3Me-*d*  3Me-*u* | 85.9 | 148.9  150.6 | 107.1  105.4 | 129.4  128.8 | 13.8  13.8 | ---- |
|  |  | 5Me-*u* |  | 139.4 | 108.2 | 140.1 | ---- | 12.6 |
|  | *udd* | 3Me-*u*  3Me-*d* | 80.6 | 148.6  152.1 | 105.7  105.5 | 133.8  128.7 | 13.9  13.5 | ---- |
|  |  | 5Me-*d* |  | 137.8 | 106.6 | 137.2 | ---- | 10.6 |
|  | ***uud*** | **3Me-*u***  **3Me-*u*** | **80.4** | **150.5**  **150.2** | **106.2**  **105.4** | **130.4**  **128.1** | **13.7**  **13.7** | **----** |
|  |  | **5Me-*d*** |  | **139.7** | **105.3** | **140.3** | **----** | **10.7** |
|  | *uuu* | 3Me-*u* | 86.2 | 152.1  151.6 | 104.6  105.9 | 129.8  128.3 | 13.9  13.8 | ---- |
|  |  | 5Me-*u* |  | 139.4 | 108.1 | 140.8 | ---- | 11.0 |
| **355** | *ddd* | 3Me-*d* | 80.2 | 149.9 | 107.5 | 128.5 | 14.1 | ---- |
|  |  | 5Me-*d*  5Me-*d* |  | 138.4  137.9 | 107.2  107.0 | 138.8  138.5 | ---- | 10.5  10.3 |
|  | ***ddu*** | **3Me-*d*** | **84.3** | **150.8** | **105.9** | **129.6** | **13.9** | **----** |
|  |  | **5Me-*u***  **5Me-*d*** |  | **138.8**  **137.5** | **107.2**  **107.0** | **144.5**  **139.4** | **----** | **11.8**  **10.9** |
|  | *duu* | 3Me-*d* | 86.7 | 152.5 | 106.2 | 131.8 | 13.9 | ---- |
|  |  | 5Me-*u*  5Me-*u* |  | 139.5  138.4 | 106.2  107.6 | 141.8  142.0 | ---- | 10.3  11.4 |
|  | *udd* | 3Me-*u* | 78.8 | 148.8 | 106.2 | 133.0 | 13.6 | ---- |
|  |  | 5Me-*d*  5Me-*d* |  | 139.5  137.7 | 106.2  106.8 | 139.1  136.6 | ---- | 11.5  10.0 |
|  | *uud* | 3Me-*u* | 84.3 | 150.5 | 105.6 | 128.9 | 13.8 | ---- |
|  |  | 5Me-*u*  5Me-*d* |  | 139.3  137.5 | 108.4  107.0 | 141.7  139.8 | ---- | 12.6  10.5 |
|  | *uuu* | 3Me-*u* | 87.1 | 152.6 | 106.0 | 129.6 | 13.8 | ---- |
|  |  | 5Me-*u*  5Me-*u* |  | 140.2  138.9 | 107.4  107.9 | 142.8  140.8 | ---- | 10.5  10.7 |
| **555** | *ddd* | 5Me-*d* | 78.7 | 138.5 | 107.1 | 139.1 | ---- | 10.6 |
|  | ***udd*** | **3Me-*u*** | **83.1** | **139.1** | **107.7** | **145.0** | **----** | **12.2** |
|  |  | **3Me-*d*** |  | **137.6**  **138.8** | **107.8**  **106.5** | **140.3**  **139.3** | **----** | **11.0**  **10.6** |
|  | *uud* | 5Me-u | 84.0 | 138.4  139.5 | 107.2  106.7 | 141.7  142.5 | ---- | 10.9  10.9 |
|  |  | 5Me-*d* |  | 139.6 | 107.0 | 141.6 | ---- | 11.2 |
|  | *uuu* | 5Me-*u* | 87.9 | 140.0 | 107.6 | 142.4 | ---- | 11.3 |

| **Table ES3:** GIAO calculated ^15^N chemical shifts (ppm) | | | |
| --- | --- | --- | --- |
| **333** *ddd* | 3Me-*d* | –175.2 | –72.6 |
| **333** *udd* | 3Me-*u* | –173.6 | –77.6 |
|  | 3Me-*d* | –171.2  –165.6 | –76.1  –79.9 |
| **333 *uud*** | **3Me-*u*** | **–173.4**  **–168.8** | **–73.6**  **–78.0** |
|  | **3Me-*d*** | **–170.6** | **–84.8** |
| **333** *uuu* | 3Me-*u* | –173.0 | –74.8 |
| **335** *ddd* | 3Me-*d* | –176.3  –176.8 | –72.6  –72.8 |
|  | 5Me-*d* | –170.8 | –69.9 |
| **335** *ddu* | 3Me-*d*  3Me-*d* | –173.8  –175.0 | –82.6  –75.8 |
|  | 5Me-*u* | –169.4 | –66.2 |
| **335** *duu* | 3Me-*d*  3Me-*u* | –173.4  –175.4 | –76.3  –77.0 |
|  | 5Me-*u* | –167.2 | –65.9 |
| **335** *udd* | 3Me-*d*  3Me-*u* | –167.1  –173.7 | –80.5  –78.1 |
|  | 5Me-*d* | –169.2 | –71.1 |
| **335 *uud*** | **3Me-*u***  **3Me-*u*** | **–168.2**  **–173.2** | **–79.2**  **–73.6** |
|  | 5Me-*d* | –165.6 | –81.6 |
| **335** *uuu* | 3Me-*u*  3Me-*u* | –173.7  –174.7 | –77.5  –74.7 |
|  | 5Me-*u* | –170.8 | –66.2 |
| **355** *ddd* | 3Me-*d* | –178.0 | –72.7 |
|  | 5Me-*d*  5Me-*d* | –171.7  –171.2 | –69.8  –69.7 |
| **355 *ddu*** | **3Me-*d*** | **–177.6** | **–76.5** |
|  | **5Me-*u***  **5Me-*d*** | **–167.9**  **–166.9** | **–69.8**  **–71.3** |
| **355** *duu* | 3Me-*d* | –174.4 | –77.1 |
|  | 5Me-*u*  5Me-*u* | –171.4  –165.1 | –69.6  –70.4 |
| **355** *udd* | 3Me-*u* | –173.8 | –78.4 |
|  | 5Me-*d*  5Me-*d* | –171.7  –162.5 | –69.9  –75.9 |
| **355** *uud* | 3Me-*u* | –176.1 | –76.7 |
|  | 5Me-*u*  5Me-*d* | –168.5  –167.9 | –65.2  –72.4 |
| **355** *uuu* | 3Me-*u* | –174.2 | –74.0 |
|  | 5Me-*u*  5Me-*u* | –172.1  –169.2 | –69.5  –66.3 |
| **555** *ddd* | 5Me-*d* | –173.5 | –70.2 |
| **555 *udd*** | **5Me-*u*** | **–168.8** | **–70.0** |
|  | **5Me-*d***  **5Me-*d*** | **–173.1**  **–167.1** | **–73.1**  **–70.3** |
| **555** *uud* | 5Me-*u*  5Me-*u* | –165.4  –171.4 | –70.5  –68.4 |
|  | 5Me-*d* | –170.1 | –73.3 |
| **555** *uuu* | 5Me-*u* | –170.3 | –68.4 |

| **Table ES4:** Free-Wilson matrix for GIAO calculated ^15^N chemical shifts | | | | | | | | | |
| --- | --- | --- | --- | --- | --- | --- | --- | --- | --- |
| No | Code | N1 | N2 | 3Me | *u* | 3Me-*u* | 3Me-*d* | 5Me-*u* | 5Me-*d* |
| 1 | **333** *ddd* | –175.2 | –72.6 | 1 | 0 | 0 | 2 | 0 | 0 |
| 2 | **333** *ddd* | –175.2 | –72.6 | 1 | 0 | 0 | 2 | 0 | 0 |
| 3 | **333** *ddd* | –175.2 | –72.6 | 1 | 0 | 0 | 2 | 0 | 0 |
| 4 | **333** *udd* | –173.6 | –77.6 | 1 | 1 | 0 | 2 | 0 | 0 |
| 5 | **333** *udd* | –171.2 | –76.1 | 1 | 0 | 1 | 1 | 0 | 0 |
| 6 | **333** *udd* | –165.6 | –79.9 | 1 | 0 | 1 | 1 | 0 | 0 |
| 7 | **333 *uud*** | –173.4 | –73.6 | 1 | 1 | 1 | 1 | 0 | 0 |
| 8 | **333 *uud*** | –168.8 | –78.0 | 1 | 1 | 1 | 1 | 0 | 0 |
| 9 | **333 *uud*** | –170.6 | –84.8 | 1 | 0 | 2 | 0 | 0 | 0 |
| 10 | **333** *uuu* | –173.0 | –74.8 | 1 | 1 | 2 | 0 | 0 | 0 |
| 11 | **333** *uuu* | –173.0 | –74.8 | 1 | 1 | 2 | 0 | 0 | 0 |
| 12 | **333** *uuu* | –173.0 | –74.8 | 1 | 1 | 2 | 0 | 0 | 0 |
| 13 | **335** *ddd* | –176.3 | –72.6 | 1 | 0 | 0 | 1 | 0 | 1 |
| 14 | **335** *ddd* | –176.8 | –72.8 | 1 | 0 | 0 | 1 | 0 | 1 |
| 15 | **335** *ddd* | –170.8 | –69.9 | 0 | 0 | 0 | 2 | 0 | 0 |
| 16 | **335** *ddu* | –173.8 | –82.6 | 1 | 0 | 0 | 1 | 1 | 0 |
| 17 | **335** *ddu* | –175.0 | –75.8 | 1 | 0 | 0 | 1 | 1 | 0 |
| 18 | **335** *ddu* | –169.4 | –66.2 | 0 | 1 | 0 | 2 | 0 | 0 |
| 19 | **335** *duu* | –173.4 | –76.3 | 1 | 0 | 1 | 0 | 1 | 0 |
| 20 | **335** *duu* | –175.4 | –77.0 | 1 | 1 | 0 | 1 | 1 | 0 |
| 21 | **335** *duu* | –167.2 | –65.9 | 0 | 1 | 1 | 1 | 0 | 0 |
| 22 | **335** *udd* | –167.1 | –80.5 | 1 | 1 | 0 | 1 | 0 | 1 |
| 23 | **335** *udd* | –173.7 | –78.1 | 1 | 0 | 1 | 0 | 0 | 1 |
| 24 | **335** *udd* | –169.2 | –71.1 | 0 | 0 | 1 | 1 | 0 | 0 |
| 25 | **335 *uud*** | –168.2 | –79.2 | 1 | 1 | 1 | 0 | 0 | 1 |
| 26 | **335 *uud*** | –173.2 | –73.6 | 1 | 1 | 1 | 0 | 0 | 1 |
| 27 | **335 *uud*** | –165.6 | –81.6 | 0 | 0 | 2 | 0 | 0 | 0 |
| 28 | **335** *uuu* | –173.7 | –77.5 | 1 | 1 | 1 | 0 | 1 | 0 |
| 29 | **335** *uuu* | –174.7 | –74.7 | 1 | 1 | 1 | 0 | 1 | 0 |
| 30 | **335** *uuu* | –170.8 | –66.2 | 0 | 1 | 0 | 2 | 0 | 0 |
| 31 | **355** *ddd* | –178.0 | –72.7 | 1 | 0 | 0 | 0 | 0 | 2 |
| 32 | **355** *ddd* | –171.7 | –69.8 | 0 | 0 | 0 | 1 | 0 | 1 |
| 33 | **355** *ddd* | –171.2 | –69.7 | 0 | 0 | 0 | 1 | 0 | 1 |
| 34 | **355 *ddu*** | –177.6 | –76.5 | 1 | 0 | 0 | 0 | 1 | 1 |
| 35 | **355 *ddu*** | –167.9 | –69.8 | 0 | 0 | 0 | 1 | 0 | 1 |
| 36 | **355 *ddu*** | –166.9 | –71.3 | 0 | 1 | 0 | 1 | 0 | 1 |
| 37 | **355** *duu* | –174.4 | –77.1 | 1 | 0 | 0 | 0 | 2 | 0 |
| 38 | **355** *duu* | –171.4 | –69.6 | 0 | 1 | 0 | 1 | 1 | 0 |
| 39 | **355** *duu* | –165.1 | –70.4 | 0 | 1 | 0 | 1 | 1 | 0 |
| 40 | **355** *udd* | –173.8 | –78.4 | 1 | 1 | 0 | 0 | 0 | 2 |
| 41 | **355** *udd* | –171.7 | –69.9 | 0 | 0 | 1 | 0 | 0 | 1 |
| 42 | **355** *udd* | –162.5 | –75.9 | 0 | 0 | 1 | 0 | 0 | 1 |
| 43 | **355** *uud* | –176.1 | –76.7 | 1 | 1 | 0 | 0 | 1 | 1 |
| 44 | **355** *uud* | –168.5 | –65.2 | 0 | 1 | 1 | 0 | 0 | 1 |
| 45 | **355** *uud* | –167.9 | –72.4 | 0 | 0 | 1 | 0 | 1 | 0 |
| 46 | **355** *uuu* | –174.2 | –74.0 | 1 | 1 | 0 | 0 | 2 | 0 |
| 47 | **355** *uuu* | –172.1 | –69.5 | 0 | 0 | 1 | 0 | 1 | 0 |
| 48 | **355** *uuu* | –169.2 | –66.3 | 0 | 0 | 1 | 0 | 1 | 0 |
| 49 | **555** *ddd* | –173.5 | –70.2 | 0 | 0 | 0 | 0 | 0 | 2 |
| 50 | **555** *ddd* | –173.5 | –70.2 | 0 | 0 | 0 | 0 | 0 | 2 |
| 51 | **555** *ddd* | –173.5 | –70.2 | 0 | 0 | 0 | 0 | 0 | 2 |
| 52 | **555 *udd*** | –168.8 | –70.0 | 0 | 1 | 0 | 0 | 0 | 2 |
| 53 | **555 *udd*** | –173.1 | –73.1 | 0 | 0 | 0 | 0 | 1 | 1 |
| 54 | **555 *udd*** | –167.1 | –70.3 | 0 | 1 | 0 | 0 | 0 | 2 |
| 55 | **555** *uud* | –165.4 | –70.5 | 0 | 1 | 0 | 0 | 1 | 1 |
| 56 | **555** *uud* | –171.4 | –68.4 | 0 | 1 | 0 | 0 | 1 | 1 |
| 57 | **555** *uud* | –170.1 | –73.3 | 0 | 0 | 0 | 0 | 2 | 0 |
| 58 | **555** *uuu* | –170.3 | –68.4 | 0 | 1 | 0 | 0 | 2 | 0 |
| 59 | **555** *uuu* | –170.3 | –68.4 | 0 | 1 | 0 | 0 | 2 | 0 |
| 60 | **555** *uuu* | –170.3 | –68.4 | 0 | 1 | 0 | 0 | 2 | 0 |

**Table ES5** Crystal data, data collection and structure refinement for compound **335**.

| Crystal data | | | |
| --- | --- | --- | --- |
| Chemical formula | C_13_H_16_N_6_ | *M*_r_ | 256.32 |
| Crystal system, space group | Triclinic, *P-1* | *Z* | 2 |
| Radiation type | Cu *K*α | μ (mm^-1^) | 0.647 |
| *a*, *b*, *c* (Å) | 8.4014(8), 8.9956(8), 10.2829(9) | α, β, γ (°) | 73.124(3), 68.239(3), 77.347(4) |
| *V* (Å^3^) | 685.23(11) | Temperature (K) | 296 |
| Data collection | | | |
| Diffractometer | Bruker *APEX*-II CCD | Absorption correction | Multi-scan (*SADABS;* Bruker, 2013) |
| *T*_min_, *T*_max_ | 0.6480, 0.7533 | *R*_int_ | 0.0420 |
| No. of measured, independent and observed [*I* > 2σ(*I*)] reflections | 19311, 2193, 1852 | 2Θ range for data collection (°) | 10.358 to 127.374 |
| Refinement | | | |
| *R*[*F*^2^ > 2σ(*F*^2^)], *wR*(*F*^2^), *S* | 0.0959, 0.2541, 1.095 | Data/restraints/param. | 2193/0/175 |
| H-atom treatment | H-atom parameters constrained | Δρ_max_, Δρ_min_ (e Å^-3^) | 0.68/-0.33 |
